# African Primary Care Research: Performing surveys using questionnaires

**DOI:** 10.4102/phcfm.v6i1.589

**Published:** 2014-05-06

**Authors:** Indiran Govender, Langalibalele H. Mabuza, Gboyega A. Ogunbanjo, Bob Mash

**Affiliations:** 1 Department of Family Medicine, University of Limpopo, Medunsa Campus, South Africa; 2 Division of Family Medicine and Primary Care, Stellenbosch University, South Africa

## Abstract

The aim of this article is to provide practical guidance on conducting surveys and the use of questionnaires for postgraduate students at a Masters level who are undertaking primary care research. The article is intended to assist with writing the methods section of the research proposal and thinking through the relevant issues that apply to sample size calculation, sampling strategy, design of a questionnaire and administration of a questionnaire. The article is part of a larger series on primary care research, with other articles in the series focusing on the structure of the research proposal and the literature review, as well as quantitative data analysis.

## Abstrait

**La recherche sur les soins de santé primaire en Afrique: réaliser des sondages à l'aide de questionnaires**. L'objectif de cet article est de fournir des directives pratiques pour la réalisation de sondages et l'utilisation de questionnaires, directives destinées aux étudiants de troisième cycle en année de Master qui mènent des recherches sur les soins de santé primaire. L'article est destiné à les aider à rédiger la section méthodologique de leur proposition de recherche et à réfléchir aux questions pertinentes relatives au calcul de la dimension de l’échantillon, à la stratégie d’échantillonnage, à l’élaboration d'un questionnaire et à son administration. L'article fait partie d'une série dédiée à la recherche sur les soins de santé primaire, et, comme d'autres articles composant cette série, traite essentiellement de la structure de la proposition de recherche, de l’étude bibliographique, et de l'analyse quantitative des données.

## Introduction

This article is part of a series on African primary care research. In this article the authors address the use of surveys and questionnaires. This is a very common study design in primary care research and the article is intended to guide primary care researchers and postgraduate students at a Masters level with regard to the development of their research proposal. Other articles in the series focus on related issues such as the structure of the research proposal and the literature review, as well as quantitative data analysis.

## Study design

The type of survey to be used should be described in the section on study design in your research proposal. For example, you may describe the design as a descriptive survey or an observational cross-sectional analytical study.

Surveys are used to obtain information on or to measure the distribution of selected characteristic(s) in a group or population of interest.^1, 2^ In simple terms, a survey encompasses any measurement procedure that involves administering a questionnaire to respondents. Questionnaires offer an objective means of collecting information about people's knowledge, beliefs, attitudes and behaviour. They can be used as the sole research instrument (such as in a cross-sectional survey) or within clinical trials and other epidemiological studies.^3, 4^

Surveys are often descriptive in nature and help to quantify the frequency with which the variables of interest occur in a population. A cross-sectional survey can be used as a type of observational study, which goes beyond simple description to analyse and compare variables in the population. For example, a cross-sectional survey may divide the population into those who are adherent and non-adherent to antiretroviral treatment and then analyses the other variables collected in order to see if any are associated significantly with this outcome. In this type of observational study a hypothesis that certain variables may be associated with a particular outcome is tested.^2^

Before committing to a survey as your study design, consider if a questionnaire is the most appropriate tool. Sometimes, a questionnaire will be appropriate within a mixed methodology study – for example, to quantify the qualitative findings of an initial exploratory phase.

## Study population

It is important to define your study population clearly before considering how you will sample from this population. Some researchers will also define a target population, which is the broadest population to which they would like to generalise their findings and then their study population, which is the population to which they actually have access. Often it is not possible to collect data from the entire study population and a representative sample must be selected, from whom data will be collected. The study population should be described in terms of people (who is included?), place (where are these people?) and, sometimes, time (over what time period?).

## Sample size calculation

If collecting quantitative data with a view to testing a hypothesis or assessing the prevalence of a disease or problem, a minimum sample size should be calculated.^5^ It is important to note that the formula for calculating sample size is different depending on whether the study is a descriptive survey or an observational cross-sectional study with analysis of comparison groups.

If you are planning a purely descriptive survey then your only concern is to estimate the variables you are interested in with sufficient precision so that you obtain a reasonably accurate picture of the situation in the larger target population. The calculation will tell you the size of the sample required to do this. If the main variable you are interested in measuring is a continuous variable, such as birth weight, then you will want a sufficient sample size in order to measure its mean value. If, however, the main variable you are interested in is a categorical variable, such as smokers and non-smokers, then you will want a sufficient sample size to measure the proportion of people with this variable. Your sample size will enable you to measure the point estimate of the variable within a certain confidence interval. A 95% confidence interval is usually chosen and, clearly, the larger the sample size the smaller the width of the 95% confidence interval and the more accurate the measurement.

To calculate sample size (*N*) for a mean you will need to decide how small you want the confidence interval (d) to be and you will also need to know the standard deviation (SD) of the variable that you are interested in. The formula to use will then be: *N* = (1.96^2^ x SD^2^)/d^2^. As an example, if you want to estimate the mean birth weight with a 95% CI of ± 50 g and the SD of birth weight is 500 g then *N* = (1.96^2^ x 500^2^)/50^2^ = 384. If the SD is not known then you should obtain it from published literature or subject experts and, if this is not possible, consider a pilot study to estimate it.

To calculate sample size (*N*) for a proportion, the same formula can be used but the SD for a proportion (*p*) is SD = *p* x (1–*p*). This implies (somewhat bizarrely) that you need to estimate the likely proportion in the population before you do your survey in order to calculate your sample size. For example, if you think the percentage of smokers is likely to be about 30% in your population then the SD = 0.3 x 0.7. If you also decide that you want the width of the 95% confidence interval to be 5% then the calculation will be *N* = (1.96^2^ x 0.3 x 0.7)/0.05^2^ = 323. If you have no idea of what the likely proportion will be then you can assume 50% for the sample size calculation.

If, however, your survey is intended to compare two groups for a particular outcome then it is no longer simply descriptive and the above sample size calculations no longer apply. In this situation you will need to consider the power of your sample size. Power is the ability to detect a difference that is actually present; it is related to the sample size, the difference that you want to detect, the variability in the data and the type of outcome variable you are dealing with (e.g. continuous or categorical). When you consult the statistician to make this calculation you will need to know what you consider to be the minimum clinically important difference between the groups and how large a risk you are willing to take that your inference will be wrong. Your inference may be wrong if you declare there is a difference, when in fact there is none (also called a type 1 or alpha error). The risk of this error is usually set at 5% or *p* = 0.05. Your inference may also be wrong if you declare there is no difference but in fact there is one (also called a type 2 or beta error). The risk of this error is often set between 10% – 20% or, conversely, a power of 80% – 90%.

Ultimately, your sample size calculation may also depend on practical issues such as your time and the feasibility of recruiting patients. Often, a compromise between accuracy or power and practicality must be reached. It is also good practice to involve a statistician in helping you with the sample size calculation. In your research proposal, you should list all of the assumptions that you have made in your calculation as described above.

## Sampling

Sampling is selecting a group from a much larger population (study population) that is similar in its trait distribution (e.g. gender, ethnicity, age, income, etc.) to the larger population. Findings made from studying the group can then be generalised to the larger population. The required *size* of the sample has been calculated above, but sampling deals with the *actual selection* of the group.^6^

Without careful planning and choosing an appropriate method for sampling it is very easy to obtain a biased sample that does not represent the population. When this happens, it is difficult to extend the findings to the larger population and the validity of the research decreases. Therefore, in order to produce meaningful results, researchers must ensure that they have chosen an appropriate sampling method to select a representative sample of participants.^6^

The main threat to representativeness is bias. A biased sample (selection or participation bias) is one which contains characteristics that are different from those of the population. This bias may happen by chance, but is usually attributable to selection bias. Selection bias is present when participants are selected systematically in a way that increases the probability of their being different from the desired study population. For example, if a researcher recruits participants from a gym, they are more likely to be healthier and fitter than the rest of the general public; or, if the larger population is made up of 51% women and 49% men, a sample (regardless of size) that is made up of 38% women and 62% men is not representative. Participation bias is any influence that affects who participated during the study, such as those people who dropped out or refused to participate.^2, 5, 6^

Various sampling techniques can be used depending on the type of research to be conducted. The two major types of techniques are probability sampling and non-probability sampling.^6^ In selecting a representative sample for a survey it is important to ensure that participants from the desired study population have an equal probability of being selected. Probability sampling is any sampling procedure that specifies the probability that each member of a population has of being selected. Probability sampling techniques include:^6, 7^

### Simple random sampling

This is when a list containing all of the population is created and used to select participants randomly. Random numbers can be generated, for example in an Excel spread sheet, to decide which people in the list to select. This random selection guarantees that each individual has an independent and equal chance of being selected. This method is very fair, unbiased and easy to carry out; it is the most common and highly-recommended technique. However, with simple random sampling there is no assurance of complete representativeness of the sample as those with rare features or conditions may be missed.^6, 7^

### Systematic sampling

This is a procedure of selection based on some simple, systematic rule such as every second or third person available or patients with odd numbers as the last digits of their medical files. The danger of this type of sampling is that there may be a hidden bias attached to the rule used to select people. For example if all male patients have a medical file ending in an odd number then only male patients will be selected in the example above. This can, however, be a practical approach to selecting people in a busy clinic where people present in no particular order.^6, 7^

### Stratified random sampling

A group selected from a population that reflects accurately certain segments of a population. In this type of sample, certain segments or traits are identified as being important to the research and the sample selected is controlled in order to ensure that those traits are accurately represented.^6, 7^ For example, in a survey of high school students you may stratify the sample by school or grade to ensure that equal numbers of students are selected from each. The students may still be selected in a random or systematic way within these strata.

### Cluster sampling

This is when the sample is gained by the random selection of clusters from a list containing all of the possible clusters within a population. This method is easy for obtaining a large and relatively random selection of participants; however, the selections lack independence. For example, a study may select community health centres randomly out of the total number of such centres available and then recruit a group of patients from the selected centres. These patients form a cluster and may share similar characteristics that are different to clusters of patients from other centres. The effect of such clustering will need to be taken into account when considering the sample size, representativeness of the sample or when making comparisons between patients in the analysis.^6, 7^

Non-probability sampling is any sampling procedure that cannot specify the probability that each member of a population has of being selected. This type of sampling is used when probability sampling is not feasible, but is generally not an acceptable approach to designing surveys. It is often used in qualitative research as described in the article on qualitative interviewing. Non-probability sampling techniques include convenience samples (including whoever happens to be available), quota sampling (a convenience sample of different subgroups such as men and women), purposive sampling (people selected on the basis of predefined criteria) and snowball sampling (people interviewed identify other people with the desired characteristics).^6, 7^

It is important to know that it is better to collect fewer questionnaires with good quality responses than high numbers of questionnaires from participants which are inaccurate or incomplete. One way of reducing the amount of inaccurate or incomplete data is to set strict exclusion criteria at the start of the research. For example, many studies exclude participants who are unable to read or write in the language of the questionnaire and those with certain physical and mental disabilities that might interfere with their ability to give informed consent or to understand the questions asked. However, research that systematically excludes hard-to-reach groups is increasingly seen as unethical and additional strategies and resources may need to be built into the study protocol at the outset. Research participants must be able to give meaningful answers (with help from a professional interviewer if necessary).^2, 3^ The inclusion and exclusion criteria for sampling must be described clearly in the proposal.

## Data collection (the questionnaire)

The data collection tool or questionnaire should be described in the research proposal and provided in full as an appendix. How the tool was obtained, adapted or developed should be described. The validity and reliability of the questionnaire should also be addressed in the proposal.

### Using standardised and validated questionnaires

Just because a questionnaire has been piloted on a few colleagues, used in previous studies, or published in a peer-reviewed journal does not mean it is either valid or reliable. Development of a valid and reliable questionnaire is necessary in order to reduce measurement error. Measurement error is the discrepancy between respondents’ attributes and their survey responses.

A valid questionnaire measures what it claims to measure. In reality, many questionnaires may not be valid. For example, a questionnaire that seeks to measure people's sexual behaviour may be less valid because it measures what they say they do, not what they actually do. Similarly, responses on questionnaires that ask health professionals how they manage particular diseases may differ significantly from actual clinical practice. An instrument developed in a different time, country, or cultural context may not be a valid measure in the group you are studying. If a valid questionnaire exists it should not be altered significantly as this affects the validity and reliability of the tool.^1, 2, 3^ Minor adaptation to the language is, however, often needed in order to make it understandable to the local culture or context.

Reliable questionnaires yield consistent results from repeated samples and different researchers over time. Differences in results come from differences between participants, not from inconsistencies in how the items are understood or how different observers interpret the responses. A standardised questionnaire is one that is written and administered so all participants are asked precisely the same questions in an identical format and responses are recorded in a uniform manner.

Standardising a measure increases its reliability.^1, 3, 5^ Three common types of reliability are inter-rater reliability (similarity between different raters using the same tool), test-retest reliability (similarity between repeated measurements on the same person) and internal consistency. Internal consistency is important for questionnaires and may be tested for by using the Cronbach's alpha statistical test, which measures how well items that are meant to measure a particular concept actually fit together in a questionnaire. For example, if the questionnaire has five questions that attempt to score how well a family physician teaches students then this test will quantify how well these questions work together to actually measure this attribute. A result of > 0.7 is considered ‘good’. Standardised questionnaires can also report on their sensitivity (how good a test is at detecting who has the condition/disease) and specificity (how good a test is at detecting who does not have the condition/disease). Screening questionnaires should have a high sensitivity.^1, 2, 3^

Before designing your own questionnaire, consider using a standardised and validated existing questionnaire. A previously-validated and published questionnaire will save time and resources; results can be compared with other studies, you need only give outline details of the instrument when writing up the work and it may be easier to get the research published. However, be careful when translating an existing questionnaire.^3, 4^ It is also possible to perform your research study on the validation of a questionnaire itself, in preparation for its actual use in the future.

Increasingly, research on health services uses standardised questionnaires designed for producing data that can be compared across studies. For example, clinical trials routinely include measures of patients’ knowledge and practice about a disease or condition, or satisfaction with health services. The validity of this approach depends on whether the type and range of closed responses reflects the full range of perceptions and feelings that people in all the different potential sampling frames might hold. Importantly, health status and quality of life instruments lose their validity when used beyond the context in which they were developed. Using one or more standard instruments alongside a short questionnaire could prevent the need to develop and validate a long list of new items.^3, 8^

### Designing your own questionnaire

It is not easy to construct your own questionnaire. With a computer it is possible to write one in a single evening provided the aim and objectives of the study are stated clearly. However developing a questionnaire that yields valid data to answer the research objectives is harder than one might think.^3, 4^ Inappropriate questions and lack of rigour inevitably lead to poor-quality data and misleading conclusions and recommendations. Using questionnaires can appear to provide quick answers to a research question, but may be inappropriately used, resulting in methodological errors.^3^

### Structure of the questionnaire

The design depends on well-defined objectives for the research to which the content of the questionnaire can be aligned.^1, 2^ Avoid questions that do not address your study objectives directly, however interesting the questions might appear. It is unethical to ask about issues not covered in your objectives and irrelevant questions make the questionnaire longer and the data will most likely not be included in your analysis. Keep the questionnaire as short and as simple as possible in order to encourage a good response rate. A typical design for a questionnaire will have the following elements:^1^

- A clear and concise title.
- An introduction that explains the aim of the research and purpose of the questionnaire.
- Clear instructions on how to complete the questionnaire.
- Questions organised into appropriate sections. Typically, the questionnaire starts with relevant demographic data (e.g. age, sex) that is neutral rather than more sensitive topics. However, the most important issues should ideally be covered early on in case the respondent fails to complete all the questions.

Questionnaires should be in a language the participant understands and, when translating a questionnaire into another language, another translator should back translate it to verify that the original meaning has not been lost.

### Types of questions

There are five types of questions that can be used: binary questions (e.g. yes/no or male/female), specific questions that do not specify options (e.g. How old are you?), multiple choice questions (in which the options should be mutually exclusive and exhaustive of all possible answers), multiple responses (where the respondent can select more than one of the options) and scaling questions (e.g. Likert or visual analogue-type scales).^9^

### Wording of the questions

Begin with a few non-threatening and engaging items. Use simple and direct language. Place the most important items strategically in the first half of the questionnaire.^1, 3^ Two words that are often used inappropriately in question stems are ‘frequently’ and ‘regularly’. A poorly-designed item might read, ‘I frequently engage in exercise’, and offer a Likert scale giving responses from ‘strongly agree’ through to ‘strongly disagree’. However, ‘frequently’ implies frequency, so a frequency-based rating scale (with options such as ‘at least once a day’, ‘twice a week’, and so on) would be more appropriate. ‘Regularly’, on the other hand, implies a pattern. One person can regularly engage in exercise once a month whereas another person can regularly do so four times a week. Other words to avoid in question stems include commonly, usually, many, some and hardly ever.^3, 10^

Avoid abbreviations and undefined terms that respondents may not be aware of, such as:

- Which type of diabetes do you have? □ T2DM □ T1DM
- What was your income last year?_____________________

Last year may mean 12 months ending today, the previous financial year or calendar year. Income could be before or after tax.

Other pitfalls in question design include too long, ambiguous, double or triple questions in one sentence, unreasonable recall period, double negatives, response choices that are not mutually exclusive, response options that are not exhaustive or questions based on unstated assumptions.^3, 10^ In multiple response questions it may be useful to require an answer to every option (e.g. yes, no, don't know) as otherwise you may not know if the option was actually considered by the respondent.

Closed-ended questions enable researchers to collect aggregated data quickly, but the range of possible answers is set by the researchers, not the respondents, and the richness of potential responses is lower. Closed-ended items often cause frustration, usually because researchers have not considered all potential responses.^3, 9^ Ticking a particular box, or even saying ‘yes’, ‘no’ or ‘maybe’ can make respondents want to explain their answer; and such free text annotations may add richly to the quantitative data. A free text box may be inserted at the end of the questionnaire (or even after particular items or sections). Note that participants need instructions (perhaps with examples) on how to complete free text items in the same way as they do for closed questions.

If open-ended questions are used or free text comments are invited, the researcher(s) must plan in advance how this qualitative data will be analysed. The use of structured questionnaires, which have a few open-ended questions for comments or clarification is not qualitative research.^3, 10^ Usually, these qualitative responses are brief and can also be categorised and quantified. Rarely, if there are more in-depth responses, they can be transcribed and analysed as in qualitative research.^11^ Adequate time, skills and resources for this analysis must be built into the study design; otherwise this will be a waste of participants’ and researchers’ time.^1, 3, 10^

Some respondents tend to agree with statements rather than disagree. For this reason, do not present items so that strongly agree always links to the same broad attitude. For example, on a patient satisfaction scale, if one question is ‘my GP generally tries to help me out’, another question should be phrased in the negative, such as ‘the receptionists are usually impolite’.^1, 3, 10^

Empirical studies have repeatedly shown that low response rates are often because participants are unable to read or follow the questionnaire. In general, questions should be short and to the point (around 12 words or less), but for issues of a sensitive and personal nature, short questions can be perceived as abrupt and threatening and longer sentences are preferred.^1, 3, 10^

### Analytical considerations

When designing your questions you should also think about the type of data that will be created and how this will be analysed. It is always better to collect continuous data whenever possible rather than categorical data as this allows you more flexibility and power in the final analysis. For example, collecting the actual age rather than asking people to tick an age category is preferred. In multiple choice questions it is preferable to not have too many categories as this will complicate the analysis, for example, when all categories must be compared in a contingency table. Please see the article on quantitative data analysis in this series.

### Piloting of questionnaires

Questionnaires may fail because participants don't understand them, can't complete them, or get offended by them. Whether the researcher has constructed his own questionnaire or is using an existing instrument, they always pilot it on participants who are representative of the study population.^1, 3^ Approval from an ethics committee is necessary for this phase.^4, 12^

Consider:

- How long do people take to complete it?
- Do any questions need to be repeated or explained?
- How do participants indicate that they have arrived at an answer?
- Do they show confusion or surprise at a particular response – if so, why?
- Short, abrupt questions may provoke short, abrupt answers.

Piloting will provide feedback to enable rephrasing of questions and will ensure a more valid and complete response. The piloting phase should include planning and testing the strategy for getting the questionnaire out and back, as well as when a reminder letter is needed. If researcher assistants are employed, they will require training.^1, 3^

## Data collection (procedures)

The way in which you will administer the questionnaire to collect your data needs to be fully described in the research proposal. You should also discuss any anticipated challenges in collecting data and how you plan to overcome them.

Survey questionnaires can be administered by personal interview (interviewer-administered), by self-administration (where the participant completes the questionnaire unassisted), or by telephone, mail (post), e-mail, cellphones or internet-based tools (such as a monkey puzzle).^1, 2^

Mail surveys using questionnaires are a cheap method of gathering information, but often suffer from a low response rate. They are ideal for large sample sizes and/or when the sample comes from a wide geographic area. These take at least eight to 12 weeks to complete. There is no possibility of interviewer bias; however, respondents cannot be probed for more information.^3, 10^

E-mail and internet surveys are very cost-effective and are the fastest method of distributing a survey. The internet user, however, does not represent the general population, although more are gaining access to the internet via their cellphones. Before undertaking an internet based surveys, consider this limitation.^1, 2^

Factors that increase the response rates:^1, 3, 4, 8, 10^

- The questionnaire is clearly designed and has a simple layout with items grouped into logical and coherent sections.
- It offers participants incentives in return for completion.
- It has been thoroughly piloted and tested.
- Participants are notified about the study in advance with a personalised invitation.
- The aim of study and means of completing the questionnaire are explained clearly.
- A researcher is available to answer questions and to collect the completed questionnaire.
- If using a postal questionnaire, a stamped addressed envelope is included.
- The participants feel they are a stakeholder in the study.
- Questions are phrased in a way that holds the participant's attention.
- The questionnaire has clear focus and purpose and is kept concise.
- The questionnaire is appealing to look at.
- The questionnaire is delivered electronically (if appropriate).
- The researchers use reminders such as phone calls or other follow-up methods.

### Advantages of written questionnaires^1, 3, 10^

- Questionnaires are very cost effective, especially when they involve large samples and many research questions.
- Questionnaires are familiar to most people and usually do not make people apprehensive.
- They reduce bias, with a uniform question presentation and no interpretation from another person.
- They are less intrusive than face-to-face or telephone interviews. Mailed questionnaires can be completed by the respondent in his own time.

### Disadvantages of written questionnaires^1, 3, 10^

- Possible low response rate especially with postal surveys. This can lower confidence in the results. Response rates may vary from 10% to 90%.
- Inability to probe responses as they allow little flexibility in the response format. This can be overcome in part by allowing space for open questions and comments.
- The lack of personal contact results in the loss of visual cues, especially when dealing with sensitive issues.
- With postal questionnaires, the intended respondent may not be the person who actually completes the questionnaire.
- Not ideal for some respondents such as illiterate people, people who have reading problems and blind people.

### Taking account of psychological and social influences

Survey research can never be completely objective. Researchers and participants are all human beings with psychological, emotional and social needs. A questionnaire means something different to participants and researchers. Researchers want data (with a view to publications, promotion, academic recognition and further income). This may lead to critical errors in piloting (e.g. piloting on friends rather than the target group), sampling (e.g. drifting toward convenience rather than random samples) and in the distribution, collection and coding of questionnaires. Staff employed to assist with a questionnaire study may not be familiar with all the tasks required to make it a success and may be unaware that covering up their ignorance or skill deficits will make the entire study unsound.^1, 5, 11^

## Conclusion

This article has covered the methodological issues involved in planning a survey and using a questionnaire, which should be described in the research proposal. The sample size calculation, sampling strategy, questionnaire design and data collection strategy have all been discussed. Designing a survey with a questionnaire that produces usable data is not as easy as it might seem. Awareness of the pitfalls is essential when planning research.
